# The analgesic efficacy of extracorporeal shock wave combined with percutaneous vertebroplasty in the treatment of osteoporotic thoracolumbar compression fractures in postmenopausal women

**DOI:** 10.1186/s12938-021-00894-4

**Published:** 2021-06-10

**Authors:** Xiaowei Liu, Hui Wang, Yang Zhang, Mingling Wang, Yujin Qiu, Xiaodong Sun, Sheng Wang

**Affiliations:** 1 Department of Spinal Surgery, Affiliated Hospital of Weifang Medical University, Weifang, 261031 China; 2 Department of Operating Room, Affiliated Hospital of Weifang Medical University, Weifang, China; 3 Department of Spine Surgery, Weifang People’s Hospital, Weifang, China; 4 Department of Anesthesiology, Weifang Medical University, Weifang, China; 5 Department of Endocrinology, Affiliated Hospital of Weifang Medical University, Weifang, China

**Keywords:** ESW, PVP, PKP, Postmenopausal woman, Osteoporotic fracture

## Abstract

**Background:**

To explore the analgesic efficacy of extracorporeal shock wave (ESW) combined with percutaneous vertebroplasty (PVP) after reduction in overextension position in the treatment of osteoporotic thoracolumbar compression fractures in postmenopausal women.

**Methods:**

The data of postmenopausal women with osteoporotic thoracolumbar compression fracture admitted in our department from January 2017 to October 2019 were analyzed retrospectively. They were divided into groups of unipedicular percutaneous kyphoplasty (U-PKP *n* = 21), bipedicular PKP (B-PKP *n* = 20), and ESW combined with PVP after reduction in overextension position (EP-PVP *n* = 18). The improvement of pain and vertebral height in three groups was compared.

**Results:**

Postoperative compression rate and Cobb angle of vertebral fractures in the three groups were all lower than those before surgery, and the differences between pre-operation and post-operation were statistically significant (*P* < 0.05). The visual analog scale (VAS) and Oswestry dysfunction index (ODI) scores of the three groups decreased significantly after the operation (*P* < 0.05). The ODI scores of the EP-PVP group in the third months after the operation were significantly improved compared with the other two groups, and the difference was statistically significant (*P* < 0.05).

**Conclusions:**

In our small-sample study, all three treatment schemes can treat osteoporotic compression fracture of thoracolumbar vertebrae in postmenopausal women, relieve pain, and improve quality of life. ESW combined with PVP after reduction in overextension position could achieve a good vertebral reduction rate and improve kyphosis, and may reduce the application of analgesic drugs.

## Background

With the increasingly severe aging trend of the population, the incidence of osteoporosis is increasing year by year among the elderly [1, 2]. Especially because of the lack of estrogen in postmenopausal women, leading to accelerated loss of sclerostin and decreasing bone strength, the proportion of osteoporotic fractures increases. It has become one of the most serious public health problems affecting postmenopausal women's physical health and quality of life [3]. Spinal fracture often occurs in the thoracolumbar vertebrae (T10–L2). Patients with thoracolumbar vertebral compression fracture seriously affect the quality of life and result in multiple complications, even causing sagittal imbalance and disability [4]. Percutaneous vertebroplasty (PVP) and percutaneous kyphoplasty (PKP) have gained the recognition of clinical doctors and society. Unipedicular and bipedicular piercing PKP are helpful to maintain the vertebral body height recovery and reduce the leakage of bone cement. However, due to the high cost, it is difficult to extensively develop in basic-level hospitals [5]. PVP after reduction in overextension position can help to restore the height of the vertebral body and restore the sagittal balance of the spine, and the bone cement dispersion is more uniform, with lower cost and relatively simple operation, which can also achieve good clinical treatment results [6]. Extracorporeal shock wave (ESW), as a local stimulation, can achieve better pain relief and greatly improve the treatment effects [7]. Thus, ESW provides a new idea to relieve osteoporosis pain.

To explore the analgesic efficacy of clinical curative effects of various treatments on osteoporotic fracture in the thoracolumbar vertebrae, this study selected 59 cases of postmenopausal women with osteoporotic fracture of thoracolumbar vertebrae in the Affiliated Hospital of Weifang Medical University. The treatment included unipedicular piercing PKP (U-PKP), bipedicular piercing PKP (B-PKP), and ESW combined with PVP after reduction in overextension position (EP-PVP). Comparing the clinical curative effect of the three treatments, we analyzed the three groups of patients with preoperative and postoperative conditions and sequentially explored the clinical efficacy and safety.

## Results

### Perioperative observation indexes

The operation time and the amount of blood loss of the B-PKP group were longer than that of the other two groups (*P* < 0.01). The surgery cost of the B-PKP and U-PKP groups was higher than that of the EP-PVP group (*P* < 0.01). There was no statistically significant difference between the three groups in terms of length of stay and amount of bone cement (*P* > 0.05), as shown in Table 1.

**Table 1:** Comparison of perioperative indicators

Item	Group			*F*	*P*
	U-PKP group (*n* = 21)	B-PKP group (*n* = 20)	EP-PVP group (*n* = 18)		
Operative time (min)	42.48 ± 6.20	56.45 ± 8.80^*^	41.94 ± 9.71	19.48	< 0.01
Hospitalization time (day)	4.00 ± 0.77	3.80 ± 0.77	3.66 ± 1.41	0.541	0.585
Blood loss (ml)	2.48 ± 0.51	4.10 ± 0.97^*^	2.42 ± 1.43	16.74	< 0.01
Bone cement content (ml)	5.29 ± 1.24	5.68 ± 0.99	5.31 ± 0.71	0.92	0.403
Surgery cost (cny)	24,966.17 ± 1637.60	26,428.69 ± 3077.93	13,316.57 ± 2344.66^*^	165.63	< 0.01

^*^P < 0.05 vs. the other 2 groups

### Comparison of postoperative complications

The X-ray is taken immediately after the operation which indicated that bone cement leakage occurred in 2 vertebrae of the B-PKP group, in 2 vertebrae of the U-PKP group, and in 4 cases in the EP-PVP group, with no statistically significant difference (*P* > 0.05). In comparison with the dispersion of bone cement in the vertebral body, the proportion of mass type and mixed type of the B-PKP group and U-PKP groups was higher than that of the EP-PVP group (*P* < 0.01). The dispersion ratio of the EP-PVP group was higher than that of the other two groups (*P* < 0.01). Postoperative follow-up found that there was one case of adjacent vertebral refracture in all three groups, with no statistically significant difference (*P* > 0.05). Patients with a refracture of the adjacent vertebral body were reoperated. There was no spinal canal leakage or spinal nerve root injury in the three groups, as shown in Table 2.

**Table 2:** Comparison of postoperative complications

Item	Group			χ^2^	*P*
	U-PKP group (*n* = 21)	B-PKP group (*n* = 20)	EP-PVP group (*n* = 18)		
Cement leakage rate [n (%)]	2 (9.52)	2 (5)	4 (22.22)	1.66	0.44
Diffusion of bone cement [n (%)]	21 (100)	20 (100)	18 (100)	25.01	< 0.01
Mass type [n (%)]	2 (9.52)	2 (10)	0 (0)^*^		
Mixed type [n (%)]	17 (80.96)	15 (75)	7 (38.89)^*^		
Diffuse type [n (%)]	2 (9.52)	3 (15)	11 (61.11)^*^		
Refracture rate [n (%)]	1 (4.76)	1 (5)	1 ( 5.56)	0.01	0.99

^*^P < 0.05 vs. the other 2 groups

### Vertebral compression rate, reduction rate, and Cobb angle

There was no statistically significant difference between the vertebral compression rate and Cobb angle among the three groups before operation (*P* > 0.05). Postoperative compression rate and Cobb angle of vertebral fractures in the three groups were all lower than those before surgery, and the differences were statistically significant between pre-operation and post-operation (*P* < 0.01). The postoperative vertebral restoration rate of the B-PKP group was higher than that of the other two groups (vs*.* U-PKP, *P* < 0.01; vs*.* EP-PVP, *P* < 0.01), as shown in Table 3.

**Table 3:** Comparison of vertebral compression rate and Cobb angle

Item	Group			*F*	*P*
	U-PKP group (*n* = 21)	B-PKP group (*n* = 20)	EP-PVP group (*n* = 18)		
Preoperative compression rate	42.12 ± 3.50	43.57 ± 3.22	41.87 ± 5.15	1.03	0.36
Postoperative compression rate	22.53 ± 1.41^t^	21.23 ± 1.56^t^	22.69 ± 5.57^t^	1.01	0.37
vertebral restoration rate	19.17 ± 3.66	22.25 ± 2.94^*^	19.18 ± 3.80	5.12	0.009
Preoperative Cobb angle	26.71 ± 1.73	26.83 ± 2.52	25.85 ± 2.27	1.12	0.34
Postoperative Cobb angle	12.97 ± 1.49^t^	12.93 ± 1.22^t^	12.51 ± 1.53^t^	0.61	0.55

^t^*P* < 0.05 vs. preoperative, ^*^P < 0.05 vs. the other 2 groups

### VAS score and ODI score

There was no significant difference in VAS score and ODI score between the three groups (*P* > 0.05). VAS and ODI scores of the three groups decreased significantly after the operation, and the difference was statistically significant compared with that before operation (*P* < 0.05). There was no statistically significant difference in VAS score between the three groups on the first day and the third months after surgery (*P* > 0.05). However, the ODI score of the EP-PVP group in the third months after the operation was significantly improved compared with the other two groups (*P* < 0.05), as shown in Table 4.

**Table 4:** Comparison of VAS score and ODI score between three groups

Item	Group			*F*	*P*
	U-PKP group (*n* = 21)	B-PKP group (*n* = 20)	EP-PVP group (*n* = 18)		
Preoperative ODI score	74.68 ± 3.76	72.97 ± 4.38	73.72 ± 4.62	0.84	0.45
Postoperative 2–3 months ODI score	28.88 ± 4.22^t^	27.63 ± 3.13^t^	21.25 ± 3.67^t*^	23.00	< 0.01
Preoperative VAS score	7.24 ± 0.70	7.35 ± 0.67	7.22 ± 0.65	0.21	0.81
Postoperative 1 days VAS score	2.90 ± 0.54 ^t^	2.85 ± 0.49^t^	2.89 ± 0.76^t^	0.05	0.96
Postoperative 2–3 months VAS score	1.62 ± 0.50^t^	1.65 ± 0.49^t^	1.33 ± 0.49^t^	2.37	0.10

^t^*P* < 0.05 vs. preoperative, ^*^P < 0.05 vs. the other 2 groups

## Discussion

Once osteoporosis fractures occur in postmenopausal women, they not only bring a decline in quality of life, but may also bring a heavy economic and ideological burden [8]. As the diagnostic level of osteoporosis increases year by year, an increasing number of patients with osteoporosis and fragility fractures receive more attention from clinicians. PVP, PKP, and other operations have been widely used and spread in China since the PVP treatment was first carried out by Galibert and Deramond in France in 1984 [9]. PKP holds in the leakage of bone cement and restores vertebral body height better than PVP, but in improving the postoperative pain and fractures, it does not have advantages. Spinal balance refers to the mutual influence of the anatomical structure and morphology of the cervical vertebra to the pelvis to maintain the stable state of the spine. Spinal imbalance, especially sagittal imbalance, causes long-term persistent back pain and even walking disorders [10]. Traditional PVP cannot effectively restore the injured vertebra height and prevent the aggravation of kyphosis in the late stage and may cause sagittal imbalance [11]. Personalized reduction in overextension position can improve the shortage of PVP. In this study, on the basis of the traditional PVP, the preoperative individualized hyperextension postural reduction was added. This individualized approach promotes the recovery of vertebral height and corrects kyphosis. On the basis of the traditional PKP, the bipedicular puncture PKP group was added to attempt to more significantly correct the local kyphotic deformity, increase the reduction rate, and try to restore the sagittal balance of the spine. The analysis of this study showed that the surgical expenses of the B-PKP and U-PKP groups were higher than that of the EP-PVP group, and the difference was statistically significant (*P* < 0.05). It was mainly related to increased balloon costs. The vertebral compression rate, reduction rate, and Cobb angle of these three groups were significantly improved after surgery, and all three treatments could improve kyphotic deformity and restore vertebral height. In comparison with the dispersion of bone cement in the vertebral body, the dispersion ratio of the EP-PVP group was higher than that of the other two groups, and the difference was statistically significant (*P* < 0.05). These may be related to the reduction of the filling pressure of bone cement, the reduced diffusion ranges of bone cement, and the reduced diffusion capacity after the balloons was opened.

Osteoporosis is a systemic disease. If osteoporosis is not treated, patients may still have chronic low back pain and body aches. Some patients even have fractures again. The treatment of osteoporosis should be started from two aspects: promoting bone formation and inhibiting bone resorption [12, 13]. Drugs that inhibit bone resorption mainly include bisphosphonates, estrogens, and calcitonins. Common drugs used to promote bone formation mainly include parathyroid hormones and anabolic steroids [14]. These drugs lack stimulation to the local fractured vertebral body or adjacent vertebral body.

ESW has been widely used in clinical practice. It can be used for urological lithotripsy, as well as having good effects in the treatment of osteopathic diseases, such as avascular necrosis of the femoral head, bone nonunion, external humerus epicondylitis, and skin ulcer [15–19]. ESW therapy is a newly noninvasive measure applied in the field of orthopedics, characterized by no trauma, low cost, high feasibility, and simple operation. It has been proved that ESW could promote new bone formation. In clinical work, a fracture with no signs of healing over 6 months is usually defined as fracture nonunion [20], and there have been a large number of studies and articles on healing fracture nonunion. Studies have shown that ESW can promote bone formation, induce angiogenesis, ease pain, and stimulate callus growth, as well as a variety of biological effects [21–23].

In this research, the postoperative pain in the three groups was significantly alleviated, and all patients got out of bed on the first day after the surgery, which significantly improved the quality of life. The U-PKP and B-PKP groups were treated with oral analgesic drugs for mild incision pain after operation or pain caused by osteoporosis and soft tissue injury. The EP-PVP group, however, was not given oral analgesic drugs, and was given ESW treatment after the surgery, which could alleviate the pain, reduce the ODI score better, alleviate the patients' pain, improve their quality of life, and reduce the application of pain-relieving medication.

However, the deficiency of this study is that the number of cases included in the ESW group was small, there was a short application time in our department, the follow-up time was short, and the therapeutic energy, optimal time, treatment frequency, and long-term clinical efficacy of ESW still need to be confirmed by further increasing the sample size.

## Conclusions

In our small-sample study, all three treatment schemes can treat osteoporotic compression fracture of thoracolumbar vertebrae in postmenopausal women, relieve pain, and improve quality of life. Bipedicular piercing PKP could be selected for a high degree of vertebral compression, for it could better improve the recovery rate of the vertebral body, improve kyphotic deformity, and possibly better prevent the occurrence of sagittal imbalance. Moreover, ESW combined with PVP after reduction in overextension position could achieve a good vertebral reduction rate and improve kyphosis, may reduce the application of analgesic drugs, and improve the patients' pain and quality of life. ESW has been widely used in clinical practice, with the advantages of non-trauma, easy operation, low cost, and almost no side effects. However, large-scale clinical trials are needed to validate our findings.

## Methods

### Clinical data

This study collected 59 patients who received surgery of thoracolumbar vertebral osteoporotic compression fracture between January 2017 and October 2019 in our spine surgery. All patients are postmenopausal women, aged 55–89 years (mean age: 71 years), and are single fracture. There were 2 cases of T10 vertebral bodies, 9 cases of T11 vertebral bodies, 23 cases of T12 vertebral bodies, 19 cases of L1 vertebral bodies, and 6 cases of L2 vertebral bodies among 59 vertebral bodies. They were randomly divided into three groups. The U-PKP group had 21 cases with unipedicular puncture, followed by bone cement injection operation. The B-PKP group had 20 cases with a bipedicular puncture, followed by bone cement injection operation. EP-PVP group has 18 cases, and the patients in this group underwent individualized hyperextension position reduction before surgery, followed by unipedicular PVP puncture surgery and then received ESW treatment after surgery. There were no statistically significant differences among the three groups in age, fracture segment distribution, injury to operation time, body mass index (BMI), and other basic data (*P* > 0.05), as shown in Table 5.

**Table 5:** Comparison of baseline data between three groups

Item	Group				*P*
	U-PKP group (*n* = 21)	B-PKP group (*n* = 20)	EP-PVP group (*n* = 18)		
Age (yrs)	70.52 ± 8.16	73.30 ± 7.23	70.56 ± 7.15	0.88	0.42
Lesion segments [n (%)]	21 (100)	20 (200)	18 (100)	2.65	0.96
T10 [n (%)]	1 (4.76)	1 (5.00)	0 (0.00)		
T11 [n (%)]	4 (19.05)	2 (10.00)	3 (16.67)		
T12 [n (%)]	7 (33.33)	8 (40.00)	8 (44.44)		
L1 [n (%)]	6 (28.57)	7 (35.00)	6 (33.33)		
L2 [n (%)]	3 (14.29)	2 (10.00)	1 (5.56)		
The time of fracture injury (day)	6.57 ± 1.36	6.40 ± 1.23	5.94 ± 1.26	1.20	0.31
BMI (kg/m^2^)	23.27 ± 1.77	22.40 ± 1.98	22.57 ± 2.05	1.15	0.32

### Inclusion criteria

(1) All were patients with primary osteoporosis, with BMD < − 2.5 SD (DXA method), and all were postmenopausal women. (2) Thoracolumbar MRI showed fresh compression fractures at 10 thoracic vertebrae to 2 lumbar vertebrae, and the degree of compression was ≥ 1/3. (3) All patients signed the informed consent and agreed to receive U-PKP operation or B-PKP operation or receive preoperative P-PVP operation and postoperative ESW treatment. (4) Clinical data are complete.

### Exclusion criteria

(1) Pathological fractures are caused by spinal tumor, spinal tuberculosis, etc. (2) Vertebral burst fractures; (3) Patients are combined with serious diseases, such as coagulation dysfunction or cardiopulmonary insufficiency, who cannot bear surgeries. (4) Osteoporosis is secondary to diabetes or hyperparathyroidism. (5) Patients have a history of taking drugs affecting bone metabolism. (6) Psychopaths and other special groups. All patients were given informed consent and approved by the hospital ethics committee.

### Anesthesia and surgical methods

All patients were anesthetized with 1% lidocaine local infiltration anesthesia, and the surgery was performed by the chief physician of the same treatment group. All surgeries were performed under digital subtraction angiography (DSA) fluoroscopy in the catheterization room.

### U-PKP group

DSA adopted anterior perspective positioning at the injured vertebral pedicle with surface display and marking. After local infiltration anesthesia, we chose the outer top of the vertebral pedicle as the puncture point. With 4.0 mm diameter of needle puncture in turn into the needle guided by X-ray. We used balloon dilatation to allow injured vertebral open reduction. The balloon pressure was set according to the principle of tolerance ability of the individual, within the range of 230–300 kPa pressure, after reset was satisfied, and a pushrod was applied to inject into injured vertebrae with bone cement at the wire-drawing stage. For the patients who still have knife edge pain and partial low back pain, which may be related to the knife edge, soft tissue injury around the fractured vertebral body and severe osteoporosis, conventional non-steroidal analgesics and intravenous zoledronate were used for anti-osteoporosis treatment after surgery.

### B-PKP group

This group underwent bipedicular puncture, bipedicular balloon open, the other surgical procedures were the same as U-PKP group. For the patients who still have knife edge pain and partial low back pain, which may be related to the knife edge, soft tissue injury around the fractured vertebral body and severe osteoporosis. Conventional non-steroidal analgesics, and intravenous zoledronate were used for anti-osteoporosis treatment after surgery.

### EP-PVP group

After admission to the hospital, with a 5–10 cm cushion underneath the thoracolumbar vertebral body (usually a thin quilt that was folded), patients underwent personalized hyperextension position reductions. Patients lay down in the operating room with cushions underneath their chest and bipedicular anterior superior iliac spine, allowing the abdomen to impend in front of the injured vertebra. The spine was allowed to stretch, and operators moderately pressed the back of the injured vertebra. For the patients who still have knife edge pain or partial low back pain, intravenous zoledronate was used for anti-osteoporosis after surgery, but non-steroidal analgesics were not used. Then, patients were sent to the medical rehabilitation clinic for moderate energy of ESW treatment (0.15 mJ/mm^2^) at 24–48 h after surgery. The second ESW treatment (0.15 mJ/mm^2^) was performed on the 30th day after surgery.

### Observation indicators

The operative time, operative blood loss, intraoperative bone cement dosage, length of stay, and hospitalization cost of the three groups were recorded and compared. After the operation, X-ray fluoroscopy with DSA can be used to collect photos, to collect and record the leakage of bone cement, the diffusion of bone cement in the vertebral body (including mass type, mixed type and diffusion type). Visual analog pain scale (VAS) and Oswestry dysfunction index (ODI) were used to evaluate the pain degree of preoperative and postoperative patients. X-ray was used to measure the height of injured vertebral body H0 in patients with compressed fracture, and the height of upper vertebral body of injured vertebra H1 and lower vertebral body of injured vertebra H2 were also measured. The height of the injured vertebra in the case of uncompressed fracture was calculated as H = (H1 + H2)/2, and the compression rate of the vertebral fracture site was calculated as = (H − H0)/H × 100%. The vertebral compression rate was measured by X-ray 1 day before and after the surgery, and the reduction rate of the vertebral overextension position was calculated (the reduction rate of the vertebral overextension position = the preoperative vertebral compression rate—the postoperative vertebral compression rate). Intraspinal leakage of bone cement, spinal nerve injury, and adjacent vertebral refracture were observed.

### Statistical methods

All data were processed by the SPSS 21.0 statistical software package. Measurement data were expressed in $\overline{\mathrm{X} }$±s. One-way analysis of variance was used for comparing the mean of multiple groups, and the least significant difference (LSD) method was used for pairwise comparison between groups. A t-test was used for comparison between the preoperative and postoperative groups. The sample size was determined by Gpower. *P* < 0.05 had statistical significance.

## Data Availability

The data used to support the findings of this study are available from the corresponding authors upon request.
